# Prevalence of acute diseases in the elderly assisted in emergency department of orthopedics

**DOI:** 10.1590/1413-78522014220200854

**Published:** 2014

**Authors:** Thiago de Angelis Guerra Dotta, Marcelo Batista Bonadio, Maria Elisabet Furlaneto, Jorge dos Santos Silva, Luiz Eugênio Garcez Leme

**Affiliations:** Universidade de São Paulo, Faculdade de Medicina, Hospital das Clínicas, São Paulo, SP, Brazil, Institute of Orthopedics and Traumatology, Hospital das Clínicas, Faculdade de Medicina, Universidade de São Paulo, São Paulo, SP, Brazil

**Keywords:** Aged, Emergency service, hospital, Orthopedics, Retrospective studies

## Abstract

**OBJECTIVE:**

To make an analysis of the care of elderly in an Emergency Department of Orthopedics with the primary objective to know the percentage of elderly treated, their conditions of origin and level of accidental conditions, and examine possible comorbidities, evolution and mortality rate.

**METHODS:**

Retrospective observational epidemiological study based on survey records of a tertiary hospital during one year (January to December 2006)

**RESULTS:**

In the year 2006 (January to December) 12,916 calls to patients older than 60 were performed.

**CONCLUSION:**

Massive attendance of the elderly population was observed, however, the vast majority related to chronic problems that do not require urgent attention. Patients requiring urgent attention suffer from trauma related to falls and are between the seventh and ninth decades of life, mostly female and requiring hospitalization for longer periods. ***Level of Evidence VI, Cases Series.***

## INTRODUCTION

The elderly are among the population groups most subject to trauma and acute disorders of the locomotor system.

The rapid growth of elderly population has resulted in a proportional increase in individuals with chronic disabilities during this phase of life. This characteristic directly affects the quality of life of the elderly, by changing their way of living and their health.

Fall is a serious public health problem among the elderly, due to its frequency, morbidity and to healthcare costs caused by this event,^1^ causing major problems in terms of public health and also regarding dedication and stress of caregivers.^2^ Along with osteoporosis it comprises the breeding ground for hip fracture, possibly the most disastrous accidental event in old age.^3^ Recent data from the international literature suggest that one in 15 elderly patients with hip fracture die during hospitalization and among those who are discharged, over 30% die in the first year post- fracture.^4^

Falls have been seen as the leading cause of accidental death in the elderly over 65 years old.^5^ Approximately one-third of seniors in this age group living in the community fall once a year.^6^ The incidence increases to 50% in Individuals over 80 years.^7^ ^,^ ^8^

The fear of falling and its direct relationship in constraint of daily and social activities are presented as the most important psychological trauma resulting from a fall in the elderly.^9^

Cohort study of 1667 elderly aged 65 years or older^10^ identified as factors related to the occurrence of fall an antecedent fracture, being a female, vision problems and difficulties in performing daily life activities.

Among physical and functional consequences of a fall are proximal femoral fractures (especially in neck or trochanter); soft tissue injuries; the inability to walk or perform daily activities; functional morbidity; fear of falling and institutionalization. These events are related to high mortality and limitation,^11^ especially after 75 years old.^12^ ^,^ ^13^ On the other hand, the assessment of causes of accidental death in the city of São Paulo among aged 60 or older puts accidents as a top cause of death (40.4%) followed by falls (30.6%).

When studying urgencies in Orthogeriatrics from the analysis of cases of ER in our environment, we find papers that show pain, bruises and fractures correspond to higher number of service demand in this population group. 

The objective of this study is to survey data on care for the elderly in a one year period at the of Orthopaedics and Traumatology Emergency Room in a tertiary university hospital, mainly seeking knowledge on the number of elderly treated, their preexisting conditions and level of accidental conditions that motivated them to search the ER. As a secondary objective, we examined possible comorbidities, evolution of treated patients, rates of hospitalization and mortality, and destination of the patients.

This is a retrospective observational epidemiological study using analysis of patient records, without identifying the subjects of study.

## MATERIALS AND METHODS

Medical records of the Emergency Orthopedics and Traumatology Emergency Department in a tertiary university hospital were collected over a one year interval, and analyzed by researchers. 

From this search, worksheets were filled with information obtained from medical records.

### Statistical treatment

Descriptive statistical analysis of the data was performed. When possible, methods for categorical or continuous data analyzes were adopted.

## RESULTS

A total of 12,916 patients older than 60 years were seen in year 2006 (January to December). Considering the total sample, 68.40% (8842 patients) were female and 31.50% (4074 patients) were male.

Among the 12,916 patients treated, 187 were admitted, representing 1.45%, and 12,729 patients (98.55%) were not hospitalized.

Regarding the duration of hospitalization, we obtained an average of 15.3 hospitalization days, standard deviation, 12.4 days; and median 11 days, with a maximum of 92 days and minimum of 4 days.

The hospitalization duration by gender, for 119 female patients, mean, 14.9 hospitalization days; standard deviation, 12.6 days; median 11 days, with maximum and minimum of 4 days. For males we obtained mean, 16 hospitalization days; standard deviation, 12.3 days; median, 13.5 days, with a maximum of 84 days and minimum of 4 days, and p = 0.4.

Regarding hospitalized patients, of 187, five progressed to death, corresponding to 2.70%. Patients discharged or transferred totalized 182 (97.30%). 

Tables 1-6 report other parameters studied:

**Table 1:** Distribution by age group.

Age (years old)	Frequency	%
<60	832	6,40%
60 - 69	6576	50,90%
70 - 79	4080	31,60%
80 - 89	1285	9,90%
90 - 99	142	1,10%
>100	1	0,00%
Total	12916	100,00%

**Table 2:** Most frequent diagnoses.

Diagnose ICD	%	Description
M796	0,24	Pain in limb
M545	0,14	Lumbar pain
M199	0,09	Non-specific arthrosis
Z048	0,02	Exam and observation, non-specific reason
S800	0,02	Knee injury
M542	0,02	Neck pain
S400	0,02	Shoulder and arm bruise
M544	0,02	Lumbago com sciatica
M259	0,02	Joint disorder, unspecified
S934	0,01	Sprain and ankle strain
S602	0,01	Contusion of other parts of wrist and hand
S423	0,01	Fracture of the humeral shaft
S700	0,01	Hip contusion
M791	0,01	Myalgia

**Table 3:** Hospitalization: distribution by age group.

Age group (years old)	Frequency	%
60 - 69	52	27,80%
70 - 79	67	35,80%
80 - 89	57	30,50%
90 - 99	11	5,90%
Total	187	100,00%

**Table 4:** Hospitalization time and age.

Age (years old)	Hospitalization time				
	Mean	SD	Median	Minimum	Maximum
Up to 75	13	7,9	11	4	38
Over 75	17,8	15,6	13	4	92
*p=0,008*					

**Table 5:** Hospitalization diagnosis.

ICD-10	N	Diagnosis
W18	88	Falls on the same level
S721	36	Pertrochanteric fracture
S720	35	Fracture of the femoral neck
S525	10	Fracture of the distal radius
M844	10	Pathological fracture not elsewhere classified
S422	9	Fracture of the upper end of the humerus
S828	7	Fracture of other parts of the leg
S822	7	Fracture of the tibial shaft
V031	6	Pedestrian injured in collision with car
W17	6	Other fall from one level to another
T814	6	Subsequent infection procedure not elsewhere classified
W10	6	Fall on and from stairs and steps
M841	6	Pseudoarthrosis

**Table 6:** Procedures during hospitalization.

Code	N	Procedure
39013138	38	Surgical reduction of femur fracture
39003124	20	Hip joint arthroplasty
39013081	12	Surgical reduction of fractures of the forearm bones
39013146	8	Surgical reduction of fractures of the knee
39018121	8	Total arthroplasty of the hip joint
38025019	7	Debridement, suture, graft
39011160	7	Surgical reduction of ankle fracture
39012131	7	Surgical reduction of femoral neck fracture
39001210	6	Withdrawal of Kirshner intraosseous wire
39011062	5	Surgical reduction of fractures of the upper end of the humerus
39011151	5	Surgical reduction of diaphyseal tibia
38008017	4	Drainage of abscess

## DISCUSSION

The demand for emergency services in our country, especially by the elderly is one undeniable reality and not dissociated from the international situation. North American authors refer to this feature as a sign of failure in the health system, in which people do not succeed in solving their health problems at other levels. However, the credibility and trust of the population certainly plays an important role in this demand, as well as self - perception of health, certainly changed in the elderly, especially the poor and the weak.

In our environment, care in the Clinical Medicine Emergency Room in a tertiary university hospital in the Emergency Room at *Hospital das Clínicas* was studied in a thesis by Barakat^16^ and revealed that the population served does not correspond to a sample of the population, concentrating older women with lower education and lower income, with a surcharge of black people over white people.

The concept of the Institution and the high power of the capacity to solve issues are relevant factors in the population's demand. 

In this paper demand is due more to the limitations of the hospital network than the primary care system.

In the case studied in the Orthopaedics and Traumatology Emergency Room in a tertiary care university hospital, the reality is strikingly different.

While the public system has several emergency departments in Clinical medicine, the availability of specialized treatment in orthopedics is more restricted. However, the same concept and capacity to solve issues variables existing in university hospitals also applies to the Orthopedics and Traumatology Emergency Room.

As musculoskeletal changes correspond to the greatest cause of physical limitation and quality of life in the elderly,^17^ it is comprehensible that the association between high prevalence of events in the elderly population, confidence in the institution and insufficient public resources explains the superlative and inadequate demand by the elderly population.

The number of older adults seen in 2006 corresponds to a large number of consultations (12,936 visits), the vast majority in the seventh and eighth decades of life. Observing, however, the profile of diagnostics it is clear that the greatest demand in the care of elderly corresponds to chronic symptomatic chronic mostly adequate to be treated at primary health units. The finding that only 1.45% of elderly patients attending the Orthopedics and Traumatology Emergency Room were hospitalized gives an estimate of this disproportion. Moreover, this relationship gives the impression of being more related to the lack of basic health care than the hospital network, unlike what can be observed in the study of Barakat.^16^

Among hospitalized patients equal age distribution between the seventh and ninth decades of life can be observed, and the number of women is almost twice the number of men, which corresponds to proportion of the general population, especially among older groups, as well as the Hospital under study.

The mean hospitalization time of 15.3 days is significantly higher than the expected average and shows a statistically significant relationship with age, but not with gender.

Hospitalizations were mainly motivated by secondary trauma to the falls with neck or trochanter femur fractures and upper limbs, the vast majority of procedures related to surgeries for treatment of these fractures.

The number of deaths (five deaths) is probably undersized for some limitation of the registry system. Other studies by our group have shown hospital mortality rate for elderly above 5% in the same institution, different from the 2.7% found in this study. 

## CONCLUSION

The data allow us to conclude that there is a massive attendance regarding the elderly population, however the vast majority of the demand is related to chronic conditions that do not require urgent attention.

The patients who actually require urgent attention, particularly from trauma related to falls, correspond to elderly people between the seventh and ninth decade, predominantly female, with a long hospital stay and high demand for support services.

Better structuring of primary care services to the elderly can reduce and adequate the demand for elderly in orthopedic and traumatology emergency care.
